# Three-dimensional mapping of cortical porosity and thickness along the superolateral femoral neck in older women

**DOI:** 10.1038/s41598-022-19866-2

**Published:** 2022-09-15

**Authors:** Aleksandar Cirovic, Ana Cirovic, Danica Djukic, Danijela Djonic, Vladimir Zivkovic, Slobodan Nikolic, Marija Djuric, Petar Milovanovic

**Affiliations:** 1 Faculty of Medicine, Center of Bone Biology, University of Belgrade, Dr Subotica 4/2, Belgrade, Serbia; 2 Faculty of Medicine, Institute of Anatomy, Laboratory of Bone Biology and Bioanthropology, University of Belgrade, Dr Subotica 4/2, Belgrade, Serbia; 3 Faculty of Medicine, Institute of Forensic Medicine, University of Belgrade, Deligradska 31a, Belgrade, Serbia

**Keywords:** Imaging, Microscopy, Anatomy, Musculoskeletal system

## Abstract

Although several studies have analyzed inter-individual differences in the femoral neck cortical microstructure, intra-individual variations have not been comprehensively evaluated. By using microCT, we mapped cortical pore volume fraction (Ct.Po) and thickness (Ct.Th) along the superolateral femoral neck in 14 older women (age: 77.1 ± 9.8 years) to identify subregions and segments with high porosity and/or low thickness—potential “critical” spots where a fracture could start. We showed that Ct.Po and Ct.Th significantly differed between basicervical, midcervical, and subcapital subregions of the femoral neck (*p* < 0.001), where the subcapital subregion showed the lowest mean Ct.Th and the highest mean Ct.Po. These cortical parameters also varied substantially with age and with the location of the analyzed microsegments along the individual’s neck (*p* < 0.001), showing multiple microsegments with high porosity and/or low thickness. Although the highest ratio of these microsegments was found in the subcapital subregion, they were also present at other examined subregions, which may provide an anatomical basis for explaining the fracture initiation at various sites of the superolateral neck. Given that fractures likely start at structurally and mechanically weaker spots, intra-individual variability in Ct.Po and Ct.Th should be considered and the average values for the entire femoral neck should be interpreted with caution.

## Introduction

Hip fracture is one of the main age-related health concerns, of which about 70% occurs in women^1,2^. Specifically, it is estimated that about 3.7 times more women above 50 years will sustain hip fracture in 2050 compared with 1990, reaching approximately 4.5 million fractures worldwide^1^. The already high costs for hip fracture treatment are also expected to rise in the next years^3^. Approximately one quarter of women with hip fracture dies within the first year, while survivors face the consequences in both physical and psychosocial dimensions of life^4,5^. Therefore, it is of great importance to understand the basis for hip fractures, so that fracture risk can be detected early and appropriate preventive and therapeutic measures can be undertaken.

Bone microarchitecture deteriorates with aging, and numerous studies have demonstrated negative effects of age on cortical and trabecular structure at the femoral neck^6–10^. If unrecognized and untreated, these age-related changes may result in hip fracture. The superolateral neck of the femur is a known fracture-initiating location in older individuals when a sudden impact from a sideways fall exceeds inherent fracture resistance of this bony region^7,11–14^. During a sideways fall, force produced by the impact is generated at the greater trochanter and afterwards transmitted to the femoral neck. Fleps et el. found that soft tissue, including muscles, absorbed most of the force, and protected the greater trochanter to a certain degree^15^. Nevertheless, the femoral neck is devoid of muscles and only relies on its bone structural properties (e.g., cortical pore volume fraction and thickness) to resist compressive stress produced by the fall.

In anatomical terms, there are three main types of hip fractures: basicervical, transcervical (midcervical), and subcapital fractures of the femoral neck^16,17^. Among these types, subcapital type is considered the most common^18,19^, while basicervical type occurs more rarely^18,20^. However, it is still not sufficiently understood why different anatomical types of the neck fractures occur even with similar fracture mechanism. It is possible that various regions of the superolateral neck cortex deteriorate differently with aging, which makes some parts more prone to initiating fracture than others. While previous studies examined the microstructural basis for hip fractures, either at the level of trabecular or cortical bone^7,13,14,21–24^, they either ignored variations within the femoral neck^24–26^, or focused on structural variation within the neck’s cross-section, usually identifying the superolateral quadrant as a critical area, but disregarding any potential variations along the superolateral neck^6,8,10,23,27^.

Since cortical pore volume fraction and cortical thickness are main determinants of bone strength^28–30^, we hypothesized that these two microstructural parameters are not uniform along the superolateral femoral neck and that there must be multiple “critical” spots—spots with high porosity and/or low cortical thickness. Therefore, the aim of our study was to map cortical pore volume fraction and cortical thickness along the superolateral femoral neck of older female individuals to identify the micro-locations in which microstructure suggests higher susceptibility to fracture.

## Results

Our microarchitectural assessment of the segments of the superolateral femoral neck showed within and between-individual variability in cortical pore volume fraction and cortical thickness along the femoral neck of older females, even within the subregions usually considered uniform (basicervical, midcervical, and subcapital) (Tables 1 and 2).

**Table 1:** Descriptive statistics of cortical pore volume fraction among 500-µm-thick cortical segments of the superolateral femoral neck.

	Cortical pore volume fraction [%]				Cortical thickness [mm]			
Name of examined region	Mean	SD	Minimum	Maximum	Mean	SD	Minimum	Maximum
Entire superolateral neck	24.1	9.6	7.4	72.2	0.24	0.06	0.09	0.36
Basicervical^a^ subregions	19.4	6.7	7.4	36.9	0.28	0.03	0.2	0.36
Midcervical^a^ subregions	22.6	7.6	8.4	61.1	0.25	0.5	0.09	0.35
Subcapital^a^ subregions	32.4	12.3	12.6	72.2	0.17	0.04	0.09	0.26
Dependence on the subregion	*P* < 0.001^x, y^				*P* < 0.001^x, y, z^			

Data are presented for the entire neck and for each subregion of the entire sample (basicervical, midcervical, and subcapital) (*N* = 14).

*Note.* N, total number of included individuals;

basicervical subregion encompassed 20% of segments close to the base of the neck, subcapital subregion encompassed 20% of cortical segments close to the femoral head, and midcervical subregion encompassed the middle 60% of cortical segments;

^x^ subcapital vs midcervical *P* < 0.05; ^y^ subcapital vs basicervical *P* < 0.05; ^z^ midcervical vs basicervical *P* < 0.05.

**Table 2:** Cortical pore volume fraction and cortical thickness per individual.

Case number	Age [years]	Femoral length [mm]	Cortical pore volume fraction [%]				Cortical thickness [mm]			
			Mean	SD	Min	Max	Mean	SD	Min	Max
P1	80	19.5	25.83	8.45	12.15	50.80	0.25	0.05	0.16	0.33
P2	62	19.5	17.38	9.11	7.36	49.62	0.26	0.03	0.16	0.30
P3	89	19.5	19.50	7.58	9.42	45.10	0.26	0.04	0.17	0.33
P4	87	19.5	30.13	16.89	9.07	72.17	0.18	0.06	0.09	0.29
P5	89	18	29.03	5.63	17.05	38.22	0.18	0.04	0.12	0.24
P6	82	22.5	28.70	7.21	15.31	47.71	0.22	0.05	0.14	0.30
P7	80	22.5	21.75	9.07	10.54	60.34	0.24	0.06	0.12	0.31
P8	85	21	22.74	5.56	12.13	37.68	0.22	0.04	0.15	0.28
P9	68	21	16.46	4.24	8.85	26.67	0.29	0.05	0.17	0.36
P10	68	18	27.03	6.27	10.58	40.51	0.25	0.05	0.13	0.31
P11	64	18	24.97	8.93	14.15	45.10	0.27	0.06	0.13	0.36
P12	64	15.5	29.30	9.80	16.22	49.76	0.19	0.04	0.13	0.29
P13	80	22.5	26.53	8.83	13.57	52.50	0.21	0.06	0.09	0.32
P14	82	19.5	17.71	5.15	10.21	35.25	0.29	0.04	0.19	0.35

Repeated-measures ANOVA between the basicervical, midcervical, and subcapital subregions showed that Ct.Po and Ct.Th significantly depended on the subregion (both *p* < 0.001). Specifically, post-hoc tests showed that the subcapital subregion had significantly higher Ct.Po than the midcervical subregion (*p* = 0.001) and the basicervical subregion (*p* = 0.001); and significantly lower Ct.Th than the midcervical subregion (*p* < 0.001) and the basicervical subregion (*p* < 0.001). Moreover, the basicervical subregion showed significantly higher Ct.Th than the midcervical subregion (*p* = 0.001).

Multiple linear regression analysis showed that segmental Ct.Po was associated with segments’ location (*p* < 0.001) and individual age (*p* = 0.03) (*R*^2^ = 0.174). Specifically, Ct.Po values increased from the base of the neck towards the femoral head, and increased with the increasing age. Segmental Ct.Th was associated with segments’ location (*p* < 0.001) and individual age (*p* < 0.001) (*R*^2^ = 0.426). Specifically, Ct.Th values decreased from the base of the neck towards the femoral head, and decreased with the increasing age.

To verify the effects of the segment’s location along the femoral neck on its Ct.Po or Ct.Th values, we also applied linear mixed-model analysis, where Ct.Po or Ct.Th was selected as a dependent variable, site (location of the cortical segment, i.e., segment number) and age group (< 80 years and ≥ 80 years) as fixed factors, and individual as a random factor. The results confirmed that site was significantly associated both with Ct.Po and Ct.Th (*p* < 0.001 for both).

**Figure 1:**
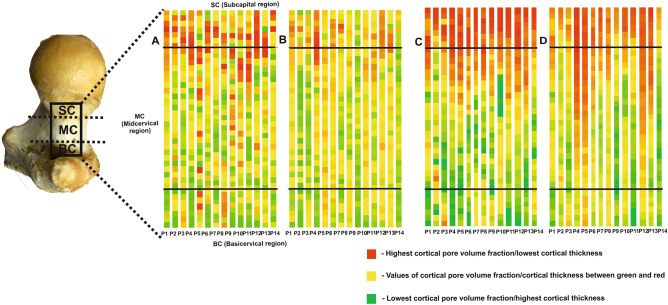
Color-coded maps of distribution of cortical pore volume fraction and thickness along the superolateral femoral neck in all individuals. Each column represents the femoral neck of one individual (P1–P14) normalized to the same length to facilitate comparisons. Color codes of cortical segments along the superolateral femoral neck range from green (the lowest porosity or highest thickness) via yellow (middle range values for both parameters) to red (highest porosity or lowest thickness). (**A** and **C**) Color codes based on the range of Ct.Po (**A**) and Ct.Th (**C**) of each individual separately. (**B** and **D**) Color codes based on the pooled range of Ct.Po (**B**) and Ct.Th (**D**) of all individuals, illustrating the distributions of Ct.Po (**B**) and Ct.Th (**D**) within individual and inter-individual differences in these parameters. Horizontal black lines are arbitrary boundaries between basicervical, midcervical, and subcapital subregions of the femoral neck.

Figure 1 illustrates considerable individual variability in cortical pore volume fraction (Fig. 1A, B) and cortical thickness (Fig. 1C, D) between the subregions, but also within the subregions (basicervical, midcervical, and subcapital). Figure 1A displays the segments of the superolateral femoral neck color-coded per value of cortical pore volume fraction and illustrates such variability along the neck. It was observed that there were potentially “critical” points (segments) in various cortical subregions within an individual, but majority of these points were located closer to the femoral head, and less often in the mid-neck, or at the base of the neck (Fig. 1A). It was also notable that there were inter-individual differences in the distribution of “critical” regions. Specifically, Fig. 1B shows color-coded distribution of cortical pore volume fraction in relation to the pooled values from all of the individuals, further illustrating inter-individual differences in cortical microstructure. Of note, although P9 and P14 have red spots in panels A and C, panels B and D show that most segments in P9 and P14 were in green to yellow range and there were no red segments, suggesting that these individuals have lower Ct.Po and Ct.Th than other individuals and possibly a lower fracture risk.

Along the length of the superolateral neck, cortical thickness varied substantially within each individual, but majority of thinner segments were located closer to the femoral head (Fig. 1C). Within the whole sample (all 500-µm-thick segments in all 14 specimens), cortical thickness varied considerably (Fig. 1D) (Table 2).

## Discussion

In this study, we demonstrated increased cortical pore volume fraction and lower cortical thickness in the subcapital subregion compared with the midcervical and basicervical subregions of the superolateral femoral neck. The midcervical subregion had lower thickness than the basicervical subregion. The subcapital and midcervical subregions particularly showed heterogeneous spatial distribution of cortical pore volume fraction. As shown by Granke et al.^31^, heterogeneous spatial distribution of cortical porosities is associated with a decrease in fracture toughness properties. Increased intra-cortical pore volume fraction is considered an essential indicator of increased risk of bone fracture^22,28,29,32,33^. Previous studies showed regional differences in cortical pore volume fraction within the cross-section of the femoral neck^8,22^. Specifically, Bell et al. divided the full circumference of the femoral neck in four regions: anterior, posterior, medial, and lateral, and showed that the lateral neck was the most porous region in women^22^. Together with other indicators of bone fragility^13,14^ and biomechanical reasons, this observation may explain why the lateral (superolateral) neck is the most common place for start of a fracture during the sideways fall^11,12,34^. Indeed, considering that cortical bone is the first to resist force caused by the impact during the fall, and is the principal contributor to the femoral neck strength^35^, increased pore volume fraction likely reduces bone strength^29^, and it was shown by mechanical testing that three quarters of the entire cortical bone strength were attributable to its porosity^36^. Therefore, the particularly increased cortical pore volume fraction in the subcapital subregion may explain why fractures are most common there; moreover, its heterogeneous spatial distribution of cortical porosities is associated with a decrease in fracture toughness properties, as shown by Granke et al.^31^.

Recently, Cirovic et al. analyzed microarchitectural parameters of two subregions of the superolateral neck in young and aged men, and reported that the basicervical subregion was significantly less porous than the subcapital region^37^. Bousson et al. investigated porosity of the medial neck in women aged between 72 and 103 years, and showed that cortical pore volume fraction varied considerably between subjects, from 4.96% to 38.87%^38^. Our study showed inter-individual differences in porosity, but also highlighted substantial variation in porosity along the entire superolateral neck of women (between 7.4% and 72.4%) (Table 1). Moreover, our results showed that average values of porosity were two-to three times lower than maximum values (depending on the region); the spots with maximum porosity (or close to maximum) are more likely the “critical” spots for fracture initiation. However, in all previous studies, the clinical implications for fracture risk were based on the assumption that the examined regions were structurally uniform^8,22,37,38^. Studies that applied µFE modeling provided some insights into the distributions of maximal principal and average tissue-level principal strain magnitudes at the entire femoral neck; however, they were based on poorer resolution and did not distinguish between the subregions of the superolateral femoral neck^39–41^.

Although subregions of the femoral neck (inferomedial, superolateral; basicervical, midcervical, and subcapital) are taken as homogenous, the results of our study highlighted substantial variability in cortical pore volume fraction and thickness along the superolateral femoral neck of an individual. There were critical points in various cortical regions within an individual, majority of which were located closer to the femoral head, and less often in the mid-neck, or at the base of the neck, especially for cortical pore volume fraction. Inter-individual variability in the distribution of “critical” segments might explain fracture initiation at different sites of the superolateral femoral neck among different individuals, assuming that the strains and stresses are similar along the femoral neck. These findings may corroborate previous observations that strain magnitudes in osteoporotic bone are less uniformly distributed^42^. Nevertheless, it was evident that the highest concentration of “weak” spots was in the subcapital region. This corresponds to the literature data about higher occurrence of subcapital fractures compared with transcervical and basicervical^43,44^. Our findings suggest that consideration of the average porosity and thickness of the entire neck or even its main subregions may not reliably reflect the actual microarchitecture of the cortical bone, partly masking the real critical spots of the femoral neck. Our study had a great advantage to analyze a number of smaller segments (each 500-µm-thick) in a representative set of individuals (older females).

Our study was limited by a cross-sectional study design. Another issue is that we did not perform any mechanical testing; in this context, to make a significant step towards quantitative prediction of failure load and location based on imaging data alone, it may be beneficial to conduct such tests and finite element studies in the future. In addition, in this study we focused only on cortical bone; nevertheless, cortical bone is the first one to accept the mechanical load and is considered crucial for the mechanical integrity of the proximal femur^45^. The location of a fracture depends also on the mechanism of fracture^46^, but it is generally believed that this is more or less similar in individuals who fall onto the greater trochanter, which puts the superolateral neck at a sudden high stress^11^. Although there are still scarce data, some FE studies showed high stress concentration at the subcapital subregion during sideways fall, and confirmed high frequency of subcapital fractures among femoral neck fractures^47–49^. Moreover, FE simulation of sideway fall showed that soft tissue absorbed some impact and reduced the force transferred through the neck^15^; nevertheless, the force is obviously sufficiently high to cause the fracture of the neck at the least resistant region. Moreover, further FE studies will have to account for the here-reported, evident, segmental variability in the femoral neck, and examine more closely whether differences in characteristics of a fall put particular strain on specific segments along the femoral neck. However, presence of structurally weak spots is a prerequisite for a fracture in any given segment area, and our study provides important clues for further evaluations of the fracture risk. Nevertheless, it should be acknowledged that cortical porosity and thickness are not the only parameters relevant for bone strength, so further studies should also examine other microarchitectural parameters (such as orientation of cortical pores) and matrix mineralization; however, as for degree of anisotropy of the cortical pores, if the selected VOI is too thin (narrow), which was the case with our 500-µm-thick segments, the value of degree of anisotropy would probably reflect the choice of the thin VOI rather than the actual spatial arrangement of the cortical pores as suggested by Tassani and Perilli for trabecular bone^50^.

Although our study provides clinically relevant data, it may be useful to perform an HR-pQCT study in a similar way to further verify clinical relevance of our findings. However, the resolution of HR-pQCT is approximately 40–80 µm, and that resolution is blind for many of the cortical pores, which is a major weakness of clinical bone microarchitecture assessment by HR-pQCT.

In conclusion, our results revealed tremendous diversity of cortical pore volume fraction as well as cortical thickness values among the segments in each examined region of the superolateral femoral neck. While the highest ratio of critical to non-critical spots was observed in the subcapital subregion, we observed a number of critical spots in other subregions as well, which offers the explanation for the microstructural basis of hip fractures in various regions of the femoral neck. Our results also emphasize that the assessments of large femoral regions that give the average values of cortical pore volume fraction or thickness may not be fully representative of the actual microarchitecture and fracture risk, as they may mask some critical spots with other “better” spots in the neighborhood. Moreover, our results suggest that more attention should be directed to heterogeneity of bone structure and heterogeneous skeletal effects of aging and diseases; considering that fracture follows the path of least resistance, the presence of particularly weak spots is more important for bone fragility than the average values.

## Materials and methods

The entire femoral neck was obtained at autopsy from 14 older women (age: 77.1 ± 9.8 years) without history of hip fracture. Neither of the included individuals had malignant diseases, or local neoplastic or degenerative diseases of the femoral neck. Neither of them used medications known to significantly deteriorate bone structure. The collection of the sample was approved by the Ethics Committee of the Faculty of Medicine, University of Belgrade (No. 29/IX-10). All methods were performed following the relevant guidelines and regulations.

By using a water-cooled, low-speed, diamond saw, the superolateral region—the region where a typical osteoporotic fracture starts—was removed from the rest of the neck. Subsequently, the entire superolateral neck was scanned using Skyscan 1172 micro-computed tomography system (Bruker microCT, Skyscan, Belgium). For scanning we used the following system parameters: 80 kV, 124 µA, exposure time of 1200 ms, aluminum and copper filter, 2 K camera binning with isotropic voxel size of 10 µm, rotation step of 0.40°, and triple frame averaging. The reconstruction of the projection images was performed in NRecon software (Bruker microCT, Belgium) on InstaRecon platform (InstaRecon, USA) with suitable thermal drift correction, misalignment compensation, Gaussian smoothing of 3, and appropriate ring artifact and beam hardening corrections.

To allow mapping of cortical pore volume fraction and thickness, we divided the entire superolateral neck length (average length 19.75 ± 2.03 mm) to a number of smaller segments. The thickness of the cortical segments was 500 µm to ensure that they are small enough to allow capturing gradients along the femoral neck, but large enough to capture a meaningfully large volume. Such a thickness was arbitrarily selected, keeping in mind to have segments that are formed by an integer number of slices (50 slices, resolution 10 µm), and provided a compromise between convenience, computer hours, and representative volumes.

**Figure 2:**
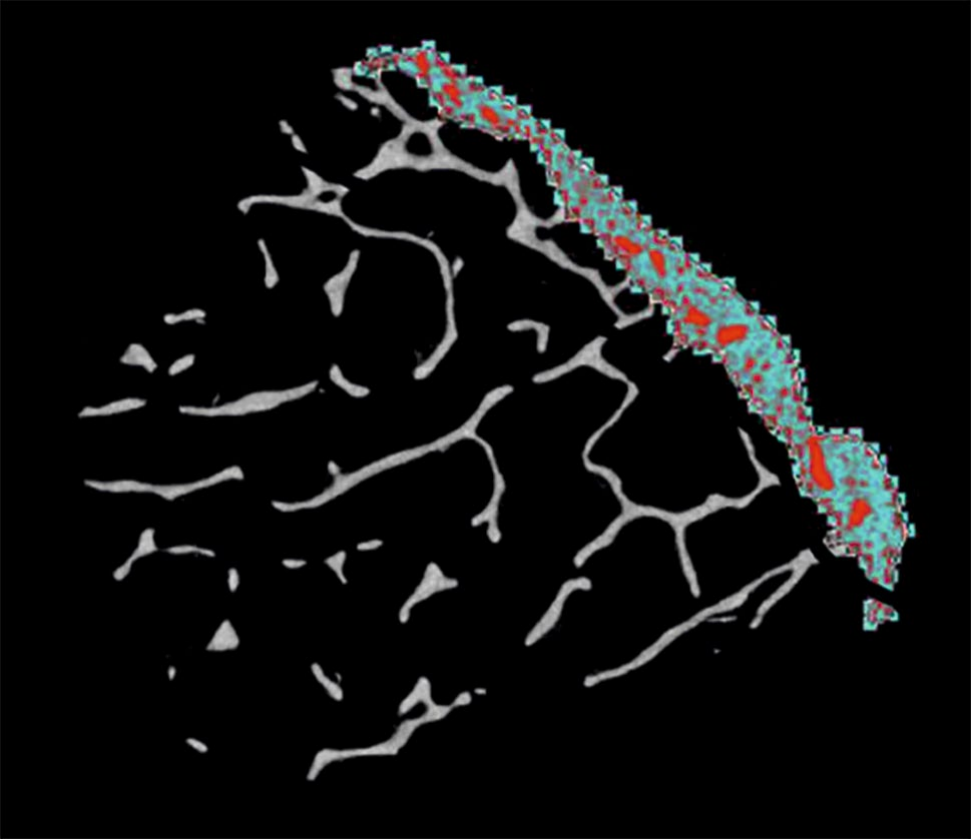
Boundaries of ROI.

In each of the segments, the cortical region of interest (ROI) was marked manually, and global threshold of 95/255 was used to distinguish between mineralized bone and marrow spaces. Cortical bone was manually and rigorously separated from trabecular bone, and transitional zone was excluded from the ROI (Fig. 2). Cortical pore volume fraction (Ct.Po, %) and cortical thickness (Ct.Th, mm) were evaluated using CT Analyzer version 1.16.4.1 Bruker, Belgium. As for cortical thickness, all cortical pores were removed based on a custom-made algorithm with Boolean operators so that cortical thickness could be evaluated automatically. Since the determination of structure thickness in 3D is based on fitting a sphere in the VOI, the thickness of the VOI lower than the actual cortical thickness would result in Ct.Th values that actually reflect the thickness of the VOI rather than the thickness of the cortex. To avoid this error, we performed 2D assessment of all slices and determined the mean Ct.Th by fitting circles in the ROI.

Based on the idea to look for “critical” spots among the segments along the neck in each individual separately, color-coded schemes of the cortical pore volume fraction and thickness values were created to visualize the distribution of “critical” points along the femoral neck (intra-individual differences). We also generated color-coded images based on the range of porosity or thickness values pooled for all individuals (inter-individual differences). Considering that the femoral neck length differed between the individuals, the data were standardized to the unit length to allow inter-individual comparisons.

SPSS version 15 was used for descriptive statistics, repeated-measures ANOVA, multiple regression analysis, and linear mixed-model analysis. Normality of data distribution was assessed by the Kolmogorov–Smirnov test. Repeated-measures ANOVA was applied to assess differences in Ct.Th or Ct.Po between the basicervical, midcervical, and subcapital regions, with post-hoc tests with Bonferroni correction for multiple testing. Multiple linear regression (method: Enter) was conducted to analyze the relationship between segmental cortical pore volume fraction or thickness values with segments’ locations and individual age, where arbitrary numbering of the segment locations started from the base of the neck. Linear mixed model was applied, where Ct.Po or Ct.Th was selected as a dependent variable, site (location of the cortical segment, i.e., segment number) and age group (< 80 years and ≥ 80 years) as fixed factors, and individual as a random factor. Results were considered significant if p values were lower than 0.05.

Microsoft Excel, version 2007, was used to prepare color-coded maps of cortical pore volume fraction and cortical thickness among the segments along the femoral neck (Fig. 1). Specifically, cortical pore volume fraction and cortical thickness values of each segment were color-coded based on their numerical values. To this end, we used the Conditional formatting option and selected Color scales; 3-Color scale was chosen, and three colors were set based on the three cutoff points of the numerical values of Ct.Po and Ct.Th as follows: red = maximum porosity, minimum thickness; yellow = 50% percentile porosity, 50% percentile thickness; green = minimum porosity, maximum thickness. Segments with porosity or thickness values between these three cutoff values were automatically assigned an appropriate color code proportionate to their value by the software. In color-coded maps (Fig. 1), each column represents the length of the femoral neck of one individual (P1–P14). Considering that the lengths of the femoral neck were not equal in each individual, they were normalized to the unit length to facilitate comparisons. Two types of color-coded maps were prepared. One type was based on the range of porosities and thicknesses of each individual, meaning that e.g. red segments mark the positions of each individual’s potentially most critical spots (high porosity; low cortical thickness) (Fig. 1A, C); nevertheless, such maps do not take into consideration the individual’s porosity or thickness values in relation to other individuals. Therefore, we also prepared color-coded maps based on the pooled range of porosities and thicknesses of all of the individuals (Fig. 1B, D), illustrating the distributions of porosity and thicknesses within individual and inter-individual differences in these parameters. Horizontal lines in the maps denote arbitrary boundaries between basicervical, midcervical, and subcapital subregions of the femoral neck (20%:60%:20%).

## Ethical review committee statement

Sample collection was based on informed consent from next of kin with approval from the Ethics Committee of the Faculty of Medicine, University of Belgrade (approval No. 29/IX-10).

## Data Availability

The data that support the findings of this study are available from the corresponding author upon reasonable request.
